# Substrate-selective mTORC1 regulation and gene dosage–dependent tissue divergence in Birt–Hogg–Dubé syndrome

**DOI:** 10.3389/fonc.2026.1848740

**Published:** 2026-07-22

**Authors:** Zhi-Lie Cao, Si-Cheng Zhao, Feng Lin, You-Huang Liu, Xu-Tao Hong, Ling-Ye Hong, Li-Qi Xu, Fei-Ping Li, Hong-Yuan Yu, Wu-Gen Yao, Wei-Ying Chen

**Affiliations:** 1 Department of Urology, Taizhou Hospital of Zhejiang Province Affiliated to Wenzhou Medical University, Enze Hospital of Hangzhou Medical College, Taizhou Enze Medical Center (Group), Taizhou, China; 2 Department of Urology, The 903rd Hospital of the Joint Logistics Support Force, Affiliated Xihu Hospital Hangzhou Medical College, Hangzhou, China; 3 Department of Ultrasound Medicine, Taizhou Hospital of Zhejiang Province Affiliated to Wenzhou Medical University, Enze Hospital of Hangzhou Medical College, Taizhou Enze Medical Center (Group), Taizhou, China; 4 Luqiao Hospital, Taizhou Enze Medical Center (Group), Taizhou, China; 5 Department of Urology, The Affiliated Jinling Hospital of Nanjing University Medical School, Nanjing, China; 6 The Fifth Hospital of Xiamen, Xiamen, Fujian, China; 7 Department of Preventive Medicine, School of Public Health, Wenzhou Medical University, Wenzhou, China; 8 Department of Urology, The 906th Hospital of the Joint Logistics Support Force, Ningbo, China; 9 Department of Urology, Linghu People’s Hospital, Huzhou, China

**Keywords:** Birt-Hogg-Dubé syndrome, FLCN, haploinsufficiency, mTORC1, renal cell carcinoma, substrate selectivity, TFEB

## Abstract

Birt-Hogg-Dubé (BHD) syndrome, caused by mutations in the tumor suppressor gene *FLCN*, has traditionally been classified as a classic “mTORopathy” characterized by global mTORC1 activation. Recent structural and multi-omic studies have fundamentally challenged this view, revealing that FLCN functions as a GAP for RagC/D to govern substrate-selective mTORC1 regulation. Synthesizing emerging evidence, we describe an integrated pathogenic framework: biallelic *FLCN* inactivation drives renal tumorigenesis via constitutive MiT/TFE nuclear accumulation and metabolic reprogramming, whereas haploinsufficiency suffices to disrupt structural integrity in the lung and skin. By reconciling the “mTORC1 paradox” through the lens of gene dosage and temporal signaling dynamics, we highlight novel therapeutic vulnerabilities targeting MiT/TFE factors and kinase rewiring, providing a rationale for organ-specific precision medicine in BHD.

## Graphical Abstract

**Figure:**
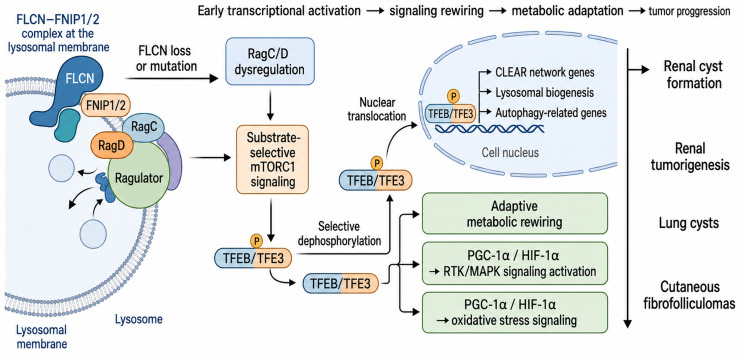
Signaling model of Birt–Hogg–Dubé syndrome (BHD).

## 1 Introduction

### 1.1 Clinical overview of Birt–Hogg–Dubé syndrome

Birt–Hogg–Dubé (BHD) syndrome is a rare autosomal dominant tumor predisposition disorder characterized by a triad of cutaneous, pulmonary, and renal manifestations (1). The syndrome was first described in 1977 by Birt, Hogg, and Dubé (2) and is classically associated with fibrofolliculomas and trichodiscomas of the face and upper trunk, multiple bilateral pulmonary cysts predisposing to spontaneous pneumothorax, and an increased lifetime risk of renal cell carcinoma (RCC) (3, 4). BHD-associated renal tumors, now increasingly recognized as FLCN-mutated tumors (FMTs), exhibit a unique histologic and molecular profile distinct from classic chromophobe RCC and oncocytoma, challenging the historical ‘hybrid tumor’ concept (5).

The disorder is caused by germline pathogenic variants in *FLCN* (folliculin), located on chromosome 17p11.2 (6). FLCN encodes a highly conserved 64-kDa protein that forms an obligate complex with FNIP1/2 and localizes predominantly to the lysosomal surface (7, 8). Initial functional studies suggested that FLCN acts as a negative regulator of mechanistic target of rapamycin complex 1 (mTORC1) (9), establishing the long-standing classification of BHD as a classical “mTORopathy”—a collective term for disorders driven by dysregulation of the mTOR signaling pathway. However, recent structural, biochemical, and multi-omic studies have substantially revised this interpretation and suggest a more nuanced role for FLCN in lysosome-centered nutrient sensing and substrate-selective mTORC1 signaling (10, 11).

### 1.2 Limitations of the classical “mTORopathy” model

Although mTORC1 dysregulation contributes to BHD pathogenesis, the concept that BHD represents a simple disorder of global mTORC1 hyperactivation does not fully explain several clinical and biological observations. In renal tumorigenesis, biallelic inactivation of *FLCN* through loss of heterozygosity (LOH) is typically required, consistent with a classical two-hit tumor suppressor model (5, 12, 13). By contrast, cutaneous fibrofolliculomas and pulmonary cysts are generally considered to arise in the setting of haploinsufficiency (14, 15). However, it is important to note that the absence of detectable LOH in pulmonary cysts has been established primarily through conventional Sanger sequencing and targeted mutation analyses. This evidence gap awaits systematic validation using modern high-sensitivity sequencing approaches. These contrasting genetic thresholds suggest that tissue-specific signaling outputs, rather than uniform mTORC1 activation, likely underlie the organ-selective manifestations of BHD.

Further complexity emerges from clinical observations. A randomized, placebo-controlled trial demonstrated that topical rapamycin did not significantly improve fibrofolliculomas (16), suggesting that canonical mTORC1 inhibition alone may be insufficient to correct certain BHD-associated phenotypes. Likewise, experimental systems have shown that canonical mTORC1 substrates such as S6K and 4E-BP1 may remain phosphorylated despite *FLCN* loss, whereas MiT/TFE transcription factors (TFEB and TFE3) become selectively dephosphorylated and accumulate in the nucleus (10, 11). These findings challenge the traditional view of uniform mTORC1 hyperactivation, supporting instead a model of substrate-selective regulation. This substrate-selective model provides a molecular basis for understanding why FLCN deficiency elicits distinct signaling outputs in different cellular contexts.

A critical caveat emerging from recent comparative analyses is that the substrate-selective model, which is characterized by preserved canonical mTORC1 activity alongside MiT/TFE nuclear accumulation, has been predominantly observed in epithelial-derived cellular systems and acute FLCN-inactivation settings. In contrast, chronic, tissue-specific, or complete biallelic loss in certain lineages may eventually bypass this substrate selectivity, leading to overt canonical mTORC1 hyperactivation. Recognizing these context-dependent boundaries is essential for accurately interpreting the divergent signaling outputs reported across different BHD models.

### 1.3 Rationale and scope of this review

Recent advances in cryo-electron microscopy, phosphoproteomics, and multi-omic tissue profiling have substantially expanded the mechanistic landscape of FLCN biology (8, 17). These studies have demonstrated that FLCN functions as a GTPase-activating protein (GAP) for RagC/D at the lysosomal surface, thereby controlling mTORC1 substrate specificity (10, 11). Loss of FLCN leads to early and persistent nuclear activation of TFEB/TFE3, followed by intermediate kinase rewiring and oxidative stress responses, and ultimately chronic metabolic reprogramming and secondary canonical mTORC1 hyperactivation in tumor contexts (17, 18).

In this review, we integrate mechanistic and clinical evidence from the past decade to propose an integrated framework that links (i) substrate-selective mTORC1 regulation, (ii) temporal progression from acute signaling disruption to chronic metabolic adaptation, and (iii) gene dosage–dependent, tissue-specific pathogenic logic. By aligning signaling dynamics with gene dosage and tissue context, we aim to provide a conceptual framework that may help explain the phenotypic heterogeneity of BHD and its therapeutic implications.

Overall, BHD should not be viewed solely as a classical mTOR-driven disorder, but rather may be better conceptualized as a lysosome-centered signaling disease in which gene dosage and tissue context dictate dominant downstream outputs. This perspective also highlights potential therapeutic strategies that extend beyond canonical mTOR inhibition toward pathway- and tissue-specific interventions.

## 2 The Rag–mTORC1 axis and substrate selectivity

### 2.1 Structural basis of FLCN function at the lysosome

FLCN is recruited to the lysosomal membrane through its obligate interaction with FNIP1/2 and the Rag–Ragulator complex, positioning the protein at a central regulatory hub of amino acid–dependent mTORC1 signaling (8, 19). This nutrient-dependent localization is coordinated with the vacuolar H+-ATPase (v-ATPase) machinery, which transmits signals of intralysosomal amino acid sufficiency to the Ragulator complex (20). Biochemical and structural studies have demonstrated that FLCN functions as a GTPase-activating protein (GAP) specifically toward RagC and RagD, promoting their GDP-bound conformation (10, 21). This GAP activity is mediated by a conserved arginine finger within FLCN that interacts with the nucleotide-binding pocket of RagC/D, underscoring its precise biochemical role (8, 19). Crucially, this regulatory mechanism contrasts with classical tumor suppressors, which directly suppress kinase activity. Instead, FLCN modulates the nucleotide state of Rag GTPases, influencing substrate recruitment.

High-resolution cryo-electron microscopy studies have clarified the architecture of the mTORC1–Rag–Ragulator megacomplex and demonstrated how the nucleotide configuration of Rag heterodimers dictates substrate docking geometry (11, 22). Structural analyses further suggest that the FLCN–FNIP complex adopts a conformation that stabilizes Rag heterodimers, facilitating proper spatial alignment required for downstream signaling (11). Within this framework, FLCN-mediated conversion of RagC/D to the GDP-bound state appears necessary for efficient lysosomal positioning and phosphorylation of MiT/TFE transcription factors. These findings indicate that FLCN primarily regulates signaling topology as opposed to functioning as a simple on/off switch for mTORC1 activity.

### 2.2 Substrate-selective mTORC1 regulation: resolving the paradox

Earlier experimental systems, particularly mouse models and certain tumor-derived lines, reported elevated phosphorylation of canonical mTORC1 substrates such as S6K and ribosomal protein S6 following *Flcn* deletion, leading to the classification of BHD as an mTOR-driven disorder (9). This interpretation was conceptually similar to the classical tuberous sclerosis complex (TSC) paradigm of global mTORC1 activation (23). However, subsequent mechanistic studies have indicated that mTORC1 outputs are not uniformly amplified after FLCN loss; instead, substrate selectivity emerges as a defining feature of FLCN deficiency.

Specifically, FLCN loss selectively impairs phosphorylation of TFEB and TFE3, resulting in their rapid dephosphorylation and nuclear accumulation, while canonical substrates such as S6K and 4E-BP1 may remain phosphorylated in acute settings (10, 17). Phosphoproteomic analyses further demonstrate that FLCN inactivation selectively alters phosphorylation at regulatory serine residues of TFEB and TFE3, while global mTORC1 kinase activity remains largely preserved (17). Mechanistically, MiT/TFE phosphorylation requires GDP-bound RagC/D, which depends on FLCN GAP activity, whereas canonical substrates are recruited through TOS motif–dependent interactions that appear less sensitive to Rag nucleotide switching (10, 22).

Consequently, the spatial recruitment and phosphorylation of MiT/TFE transcription factors are strongly dependent on the RagC/D nucleotide state controlled by FLCN (10, 24). This distinction explains the so-called “mTORC1 paradox,” wherein FLCN-deficient cells display preserved or even enhanced phosphorylation of classical mTORC1 targets yet exhibit profound transcriptional activation of MiT/TFE programs. These results are not contradictory; rather, they support a substrate hierarchy model in which MiT/TFE factors constitute a uniquely FLCN-dependent branch of mTORC1 signaling. The pathological consequence of this selective dysregulation is the constitutive nuclear accumulation of TFEB and TFE3, which drives aberrant lysosomal biogenesis, metabolic reprogramming, and eventually the renal cystogenesis and tumorigenesis characteristic of BHD syndrome (12, 25).

This substrate selectivity has been independently validated by recent studies demonstrating that active RagC, but not RagA, is necessary and sufficient to recruit TFE3 to lysosomes for mTORC1-mediated phosphorylation (26). Furthermore, the discovery of a “Rag dimer code” has added critical mechanistic nuance: RagD preferentially promotes TFEB/TFE3 phosphorylation, whereas both RagC and RagD support S6K phosphorylation (27). These findings profoundly refine our understanding of FLCN GAP activity, demonstrating that differential Rag GTPase pairing dictates substrate hierarchy at the lysosomal surface.

**Figure 1:**
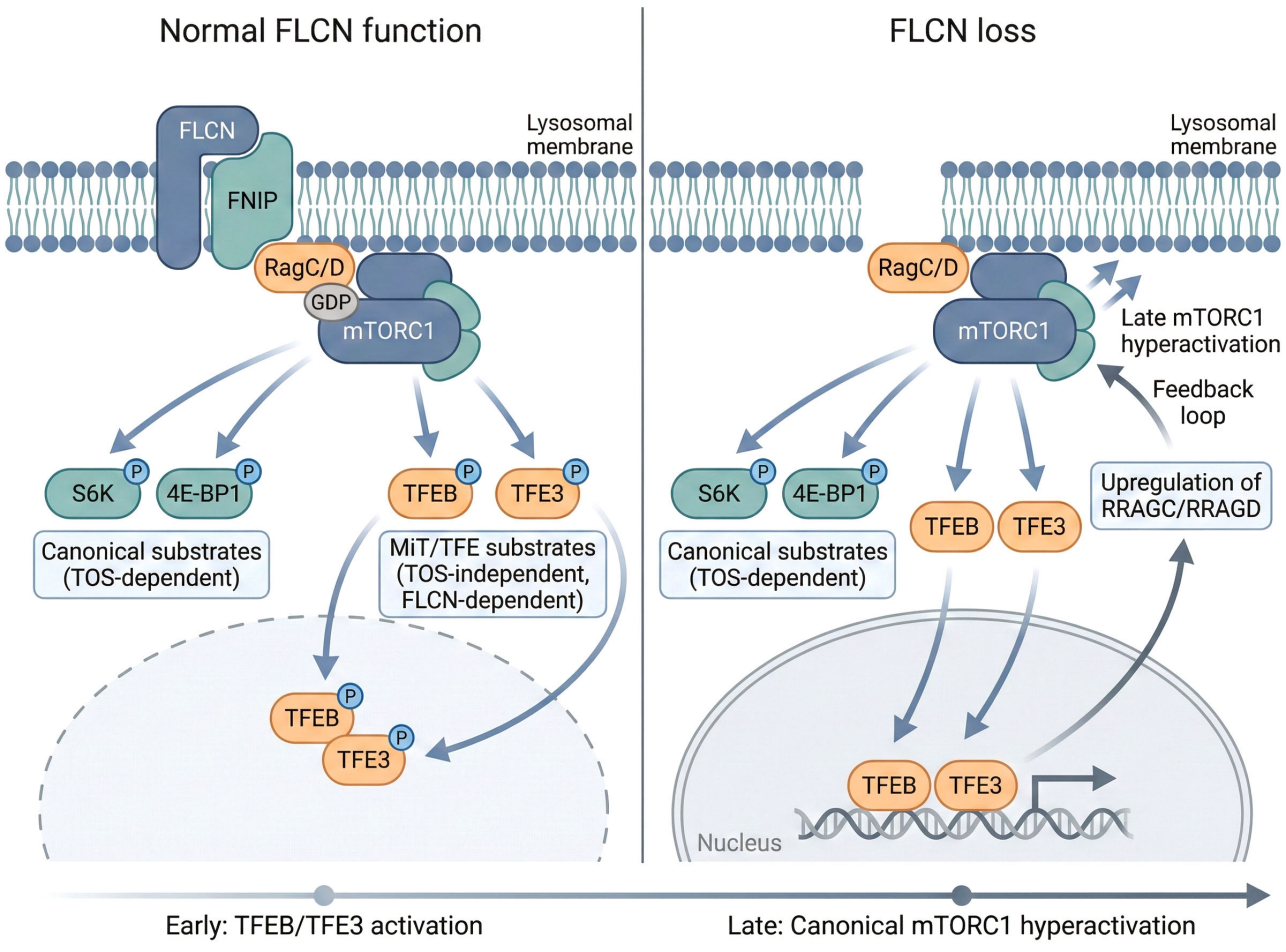
Resolution of the “mTORC1 paradox”: substrate-selective signaling following FLCN loss. Model illustrating substrate-selective mTORC1 signaling in the presence and absence of FLCN. Under physiological conditions, the FLCN–FNIP complex acts as a GAP for RagC/D, enabling recruitment of mTORC1 to the lysosome and phosphorylation of both canonical substrates (S6K, 4E-BP1) and non-canonical MiT/TFE transcription factors (TFEB/TFE3). Upon FLCN loss, mTORC1 retains the capacity to phosphorylate canonical substrates through TOS motif–dependent recruitment; however, phosphorylation of TFEB/TFE3 is selectively impaired. Consequently, TFEB/TFE3 translocate to the nucleus, activating lysosomal, metabolic, and Rag GTPase gene programs. Sustained transcriptional upregulation of RRAGC/RRAGD establishes a delayed positive feedback loop that eventually drives canonical mTORC1 hyperactivation in advanced disease stages. The temporal sequence emphasizes that MiT/TFE activation represents an early, direct biochemical consequence of FLCN deficiency, whereas robust canonical mTORC1 hyperactivation emerges later during tumor progression.

To reconcile the apparent inconsistencies between preserved canonical mTORC1 activity and robust activation of MiT/TFE transcription factors following FLCN loss, a substrate-selective regulatory framework has emerged: FLCN does not function as a global inhibitor of mTORC1; instead, it regulates the accessibility of specific substrates through Rag GTPase nucleotide states. This conceptual shift is illustrated in **Figure 1**, which summarizes how FLCN loss selectively disrupts MiT/TFE phosphorylation while sparing canonical mTORC1 outputs, thereby resolving the so-called “mTORC1 paradox”.

### 2.3 Feedback amplification and delayed canonical mTORC1 activation

Persistent nuclear TFEB/TFE3 activity induces transcription of lysosomal genes, autophagy regulators, and components of the Rag machinery, including *RRAGC* and *RRAGD*, establishing a transcriptional amplification loop (10, 28). This autoregulatory process is part of the coordinated lysosomal expression and regulation (CLEAR) network, which becomes profoundly and constitutively active upon *FLCN* loss (10, 29). Such a feed-forward circuit may gradually alter the relative abundance of Rag GTPases and other lysosomal signaling components, thereby reshaping mTORC1 dynamics over time.

Comparative analyses between acute CRISPR-engineered renal epithelial models and established tumor lines suggest that strong phosphorylation of canonical substrates (S6K, 4E-BP1) is more consistently observed in chronic or late-stage contexts (17, 18). Transcriptomic and proteomic analyses of FLCN-deficient systems further support this delayed response, demonstrating that while acute deletion primarily triggers MiT/TFE transcriptional signatures, prolonged deficiency leads to compensatory kinase rewiring and subsequently elevated canonical mTORC1 signaling (17, 18). Together, these observations support a temporal interpretation in which immediate MiT/TFE activation represents the primary biochemical consequence of FLCN loss, whereas robust canonical mTORC1 hyperactivation may emerge secondarily as part of long-term adaptation and tumor progression. These findings collectively indicate that FLCN primarily governs mTORC1 substrate hierarchy and signaling dynamics, rather than acting as a direct inhibitor of mTORC1 catalytic activity.

**Figure 2:**
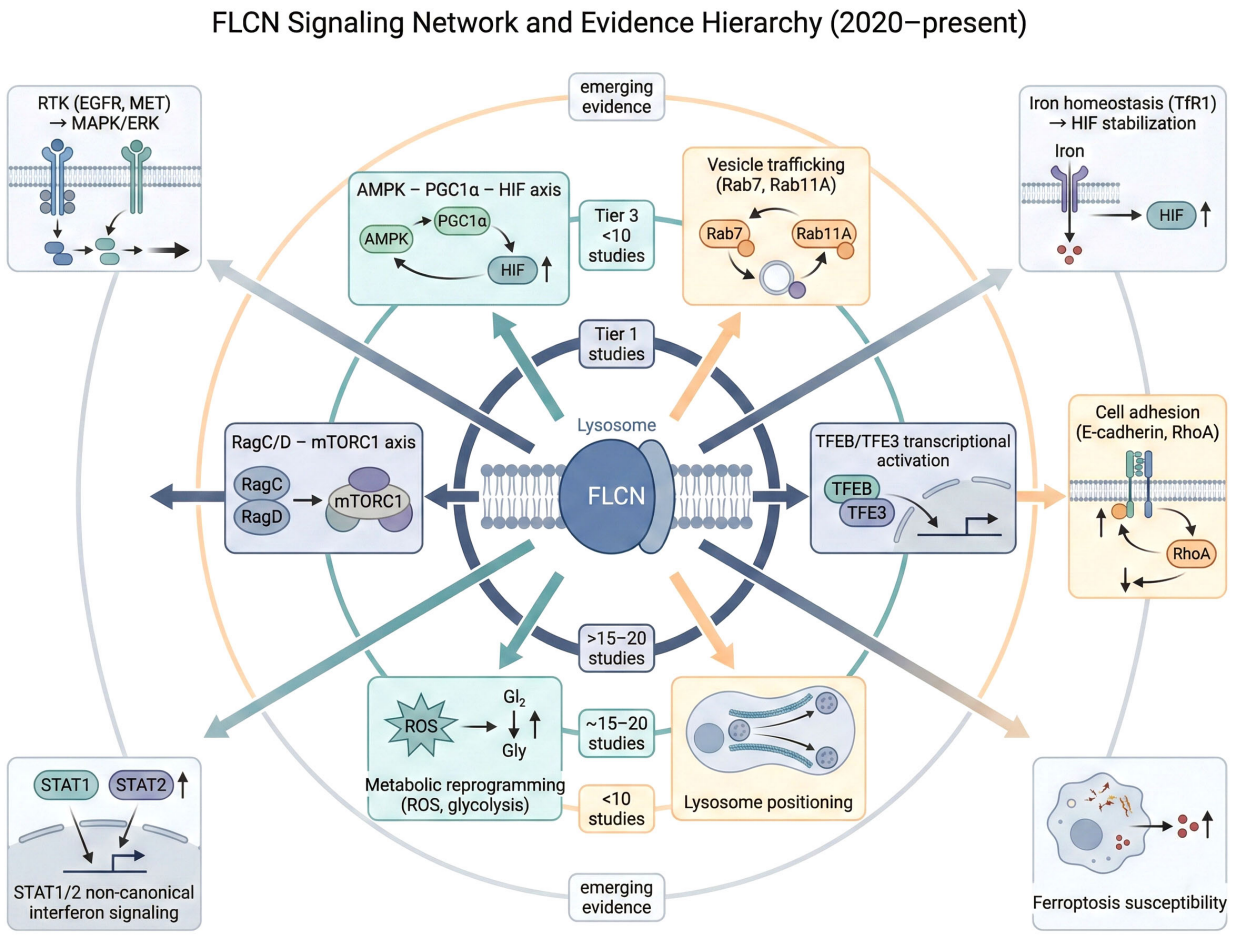
FLCN signaling network and evidence hierarchy (2020–present). Schematic overview of the major signaling axes regulated by folliculin (FLCN) based on studies published from 2020 onward. FLCN localizes to the lysosomal membrane and functions as a GTPase-activating protein (GAP) for RagC/D, thereby coordinating mechanistic target of rapamycin complex 1 (mTORC1) substrate specificity. Pathways are organized according to relative evidence density in the recent literature. Tier 1 includes the Rag–mTORC1 axis and MiT/TFE (TFEB/TFE3) transcriptional regulation, which are supported by the largest number of mechanistic and structural studies. Tier 2 encompasses AMPK–PGC1α–HIF–mediated metabolic reprogramming. Tier 3 includes vesicular trafficking, lysosomal positioning, and adhesion-related signaling. Tier 4 represents emerging or context-dependent pathways, including RTK/MAPK dependency, STAT1/2-mediated non-canonical interferon signaling, and iron homeostasis–HIF stabilization. Line thickness reflects relative study frequency rather than quantitative signaling strength. This hierarchy highlights the central lysosomal role of FLCN and the progressive expansion of its functional landscape.

Beyond individual pathways, recent studies have begun to delineate a broader signaling network downstream of FLCN, integrating transcriptional, metabolic, and kinase-level adaptations. However, the strength of evidence supporting these pathways varies considerably, ranging from well-established genetic models to emerging multi-omic and phosphoproteomic observations. **Figure 2** provides an integrated overview of the FLCN signaling network together with an evidence hierarchy, distinguishing firmly established mechanisms from emerging or context-dependent findings. As illustrated in **Figure 2**, the current evidence hierarchy places the Rag–mTORC1 axis at the apex of FLCN signaling, whereas RTK/MAPK dependencies represent emerging, context-specific vulnerabilities.

Further adding to this dynamic complexity, a recent study identified an mTORC1-mediated negative feedback loop in which constitutive mTORC1 activity impairs FLCN: FNIP2 lysosomal localization, resulting in paradoxical TFEB hypophosphorylation (30). This feedback mechanism highlights the intricate spatial and temporal autoregulation within the FLCN–mTORC1 axis.

Crucially, it must be emphasized that this three-stage temporal model currently represents a conceptual framework derived from cross-sectional comparisons. The proposed progression from immediate MiT/TFE activation to intermediate kinase rewiring and ultimately late canonical mTORC1 hyperactivation is inferred from comparisons between acute FLCN-loss systems and established tumor-derived lines. To date, no longitudinal study has directly tracked these signaling states within a single experimental system.

## 3 The TFEB/TFE3 transcriptional axis and downstream reprogramming

### 3.1 Immediate nuclear activation following FLCN loss

One of the most consistently reported molecular consequences of FLCN inactivation is the nuclear translocation of MiT/TFE family transcription factors, particularly TFEB and TFE3 (10, 25). Their nuclear accumulation is considered a central regulatory event because these transcription factors coordinate broad transcriptional programs involved in lysosomal biogenesis, autophagy, and metabolic adaptation (31). In newly engineered diploid renal epithelial cells, acute CRISPR-mediated deletion of *FLCN* leads to rapid dephosphorylation of TFEB/TFE3 and their redistribution to the nucleus, even under nutrient-replete conditions (17). Beyond nutrient signaling, cellular stress pathways such as oxidative stress also promote TFEB/TFE3 dephosphorylation via phosphatase-mediated mechanisms, indicating that multiple upstream inputs converge on this transcriptional node (32).

*In vivo* data further substantiate a causal role for MiT/TFE activation in renal pathology. Kidney-specific *Flcn* knockout mice display pronounced nuclear TFEB/TFE3 accumulation and develop rapidly progressive cystic disease (12). Genetic studies further support a functional relationship between FLCN loss and MiT/TFE activation, as truncating or frameshift mutations in FLCN frequently correlate with elevated MiT/TFE transcriptional activity in early disease models (12). Crucially, deleting *Tfeb* in this context significantly reduces cyst burden and extends survival, underscoring the functional necessity of MiT/TFE activation in early cystogenesis (12).

### 3.2 Transcriptional and metabolic programs driven by MiT/TFE factors

Transcriptomic profiling of FLCN-deficient cells reveals broad upregulation of lysosomal biogenesis genes, autophagy regulators, and metabolic enzymes under MiT/TFE control (25, 33). In renal epithelial systems, MiT/TFE activation correlates with enhanced glycolytic activity, induction of angiogenic mediators, and expression of genes involved in metabolic remodeling (18). Parallel mechanisms have been characterized in extra-renal tissues. For example, FLCN deletion in breast cancer models has been reported to induce a TFE3-dependent metabolic shift resembling the Warburg phenotype, accompanied by increased glycolytic intermediates and pro-angiogenic signaling (33). These transcriptional outputs align with metabolic and vascular features reported in BHD-associated renal tumors, including altered glucose utilization and increased vascularization.

While MiT/TFE activation upregulates autophagy initiation genes, FLCN loss may concurrently impair autophagosome-lysosome fusion or lysosomal function, resulting in the net accumulation of autophagosomes instead of promoting enhanced flux (34). Additionally, the altered spatial distribution of lysosomes following *FLCN* loss may interfere with intracellular vesicular trafficking, including the recycling of key metabolic receptors, further complicating the cellular metabolic landscape (35). The distinction between transcriptional induction of autophagy-related genes and effective completion of vesicular maturation may help reconcile conflicting reports regarding autophagic activity in FLCN-deficient systems.

### 3.3 Crosstalk with STAT1/2 and RTK/MAPK signaling

Beyond direct lysosomal gene induction, MiT/TFE activation intersects with additional signaling pathways that influence disease trajectory. *FLCN* deficiency in renal epithelial cells triggers the induction of a non-canonical, interferon-independent STAT1/2 transcriptional program (36). Functional studies suggest that this STAT1/2 transcriptional signature may exert modulatory effects on cell growth, potentially counterbalancing proliferative signals driven by TFE3 activation.

Parallel phosphoproteomic profiling has identified early hyperactivation of receptor tyrosine kinases (including EGFR, MET, and EPHA2) and downstream MAPK signaling in FLCN-deficient renal epithelial cells (17). Proteomic analyses further indicate that components of these kinase cascades and associated molecular chaperones, including Hsp90, contribute to the stabilization of signaling networks within FLCN-deficient cells (37). Moreover, the structural integrity of primary cilia, which often serve as signaling hubs for RTK and mTOR pathways, is frequently compromised following *FLCN* loss, further amplifying aberrant kinase activities (38, 39). Critically, these kinase alterations are detectable prior to overt tumorigenic transformation and may cooperate with MiT/TFE-driven transcriptional programs during early disease progression. Collectively, FLCN loss drives a dual MiT/TFE and MAPK activation program in which TFEB/TFE3 nuclear accumulation and early RTK-MAPK hyperactivation converge to sustain a feed-forward signaling circuit (12, 17). Furthermore, recent comparative transcriptomic profiling has confirmed that BHD-associated renal tumors possess a distinct molecular profile relative to sporadic chromophobe renal cell carcinomas, supporting their classification as a separate molecular entity (40).

These observations collectively indicate that MiT/TFE activation represents an early transcriptional driver following FLCN loss, whereas secondary signaling rewiring, including STAT1/2 modulation and RTK/MAPK activation, contributes to subsequent evolution of cellular phenotypes. The ultimate signaling outcome likely depends on gene dosage, tissue context, and the duration of FLCN deficiency. Further clarification of these secondary signaling interactions, particularly those occurring across renal, pulmonary, and cutaneous tissues, remains an important objective for future mechanistic studies (41, 42).

## 4 Spatial organization of lysosomes and nutrient signaling

Experimental insights into FLCN function have been derived from a spectrum of model systems that differ in their ability to capture gene dosage effects, tissue specificity, and temporal progression. While reductionist systems such as engineered cell lines are well suited for defining acute signaling events, *in vivo* and organoid models are required to interrogate chronic adaptation and tissue-dependent phenotypes. It is particularly worth highlighting that these systems inadequately capture the differential effects of haploinsufficiency versus biallelic FLCN loss, which drive organ-specific BHD manifestations. As outlined in **Table 1**, the biological scope and limitations of these models vary considerably, providing an important framework for interpreting context-dependent differences in mTORC1 signaling, MiT/TFE activation, and lysosomal behavior (5, 12, 14, 15, 17, 18, 36, 40, 43–48). This context is particularly relevant for understanding the spatial regulation of lysosomes discussed below.

**Table 1:** Experimental model systems used in FLCN/BHD research: biological scope and limitations.

Model system	Genetic strategy	Tissue context	Key questions addressed	Major strengths	Major limitations	Key references
Established tumor cell lines (e.g., UOK257)	Endogenous biallelic loss; WT add-back rescue	Human RCC	Tumor maintenance; metabolic rewiring; RTK/MAPK dependency; drug sensitivity (mTOR, EGFR/MAPK inhibitors)	Isogenic control pairs; high reproducibility; suitable for drug screening	Single-line bias; accumulated background mutations; not suitable for early tumor initiation studies	Hong et al., 2010 (43); Glykofridis et al., 2022 (17); Di Malta et al., 2023 (12)
Normal renal epithelial lines (RPTEC/TERT1)	Acute CRISPR/Cas9 knockout	Human proximal tubule	Immediate TFEB/TFE3 nuclear translocation; early kinase rewiring; STAT1/2 activation; mTORC1 timing	Clean diploid background; ideal for phosphoproteomics and RNA-seq	No long-term adaptation; lacks *in vivo* microenvironment	Glykofridis et al., 2021 (36); Glykofridis et al.2022 (17)
Normal epithelial lines (HK2; BEAS-2B)	shRNA/siRNA knockdown (acute vs chronic)	Kidney (HK2); Lung (BEAS-2B)	Chronic metabolic adaptation (PGC1α/HIF); cell-cycle changes; lung-specific miRNAs	Enables temporal comparison (acute vs long-term loss)	Incomplete depletion; RNAi off-target effects; 2D culture artifacts	Jones et al., 2025 (18); Min et al., 2020 (44)
Patient-derived primary cells (PMCs)	Germline heterozygous mutation	Human pleura	Haploinsufficiency effects; E-cadherin–LKB1–AMPK axis; apoptosis; mechanobiology	Authentic monoallelic context; high clinical relevance	Poor viability; early senescence; limited scalability	Okamoto et al., 2021 (14)
Kidney-specific mouse KO (Flcn^flox/flox; Ksp-Cre)	Conditional biallelic deletion	Mouse distal tubule/collecting duct	*In vivo* cystogenesis; TFEB/TFE3 dependency; mTORC1 hyperactivation	Organ-level pathology; genetic rescue experiments feasible	Early lethality (P22–30); cannot model long-term tumor progression	Baba et al., 2008 (45); Di Malta et al., 2023 (12)
Heterozygous/tissue-specific mouse models	Germline +/-; mesenchymal deletion	Systemic; lung mesenchyme	Two-hit tumor evolution; lung developmental defects; Wnt signaling	Recapitulates age-dependent tumor onset; models haploinsufficiency	Lung phenotype not fully matching human subpleural cysts	Chu et al., 2020 (46)
Xenograft models	Implantation of FLCN-null cells	Immunodeficient mouse	*In vivo* tumor maintenance; drug response validation	Confirms genetic dependencies *in vivo*	No immune system; ectopic site; limited organ specificity	Di Malta et al., 2023 (12)
Non-mammalian models (yeast, C. elegans, zebrafish)	Gene knockout; mutagenesis	Whole organism	Protein stability; metabolic stress; conserved pathways	High-throughput functional screening	Species divergence; limited translational relevance; zebrafish heterozygotes lack BHD phenotype	Clausen et al., 2020 (47); van Steensel et al., 2022 (15)
Patient-derived clinical specimens (FFPE, NGS, scRNA-seq)	Natural tumors	Human kidney/lung/skin	Biomarker validation; heterogeneity; mutational profiling	Highest clinical relevance	Static endpoint; no dynamic signaling; FFPE degradation artifacts	Gupta et al., 2025 (5); Jikuya et al., 2023 (40); Argani et al., 2025 (48)
Organoid models (kidney; lung; skin)	CRISPR/Cas9 KO in iPSC-derived or ASC-derived organoids; patient-derived organoids	Patient-genotype 3D tissues	Tissue architecture; cell-cell interactions; chronic signaling adaptation	Preserves tissue architecture; patient genotype; bridges in vitro/in vivo	Variable reproducibility; limited throughput; cost; immature protocols for some tissues	Freedman et al., 2015 (49); Li et al., 2022 (50)

As of this writing, no BHD-specific organoid study has been published to date. The Organoid row therefore references methodologically relevant kidney organoid studies from adjacent hereditary renal diseases (autosomal dominant polycystic kidney disease, patient-derived renal cell carcinoma) that are conceptually transferable to FLCN-deficient systems.

### 4.1 Lysosomal positioning as a regulatory node in mTORC1 signaling

It should be noted at the outset that several mechanistic claims in this section rely on indirect or correlative evidence, and direct FLCN-specific demonstration of the proposed relationships is limited in many cases. Where strong FLCN-specific data are unavailable, we have used hedging language (e.g., “may contribute,” “has been linked to,” “potentially contributing”) to reflect the preliminary nature of these observations. Readers should interpret the discussion of lysosomal spatial regulation in BHD as a framework for hypothesis generation and for guiding future experiments, rather than as a definitive mechanistic account. Direct experimental validation, including live-cell imaging of lysosomal dynamics in FLCN-deficient cells, quantitative assessment of Ragulator and mTORC1 recruitment to the lysosomal surface, and rescue of spatial phenotypes by FLCN re-expression, will be required to establish causal relationships between FLCN loss and lysosomal mispositioning.

In addition to its biochemical role as a GAP for RagC/D, FLCN has been implicated in the spatial organization of lysosomes, a cellular feature increasingly recognized as an important determinant of mTORC1 signaling dynamics. Under nutrient-replete conditions, lysosomes are typically distributed toward the cell periphery, where they participate in active nutrient sensing and signaling processes. During nutrient deprivation, lysosomes undergo dynein-dependent retrograde transport toward the perinuclear region, a redistribution that contributes to the suppression of mTORC1 signaling and activation of autophagy (51). Evidence suggests that FLCN participates in coordinating these positional transitions.

Loss of FLCN has been linked to alterations in lysosomal positioning, often leading to enhanced perinuclear clustering even under nutrient-rich conditions. This spatial mislocalization may contribute to defective amino acid sensing by the Rag–mTORC1 machinery. Consequently, nutrient-derived signals may not be efficiently relayed to downstream signaling components, potentially contributing to the signaling dysregulation observed in FLCN-deficient cells. Recent imaging-based analyses further suggest that altered lysosomal positioning affects the assembly of signaling complexes on the lysosomal membrane, including Ragulator and mTORC1 components (52, 53). These observations support a model in which FLCN contributes both to the biochemical regulation of Rag GTPases and to the spatial organization of lysosomal signaling platforms.

### 4.2 Cytoskeletal interactions and vesicular transport

Lysosomal positioning is tightly linked to cytoskeletal transport systems involving microtubules, kinesin motors, and dynein complexes. Emerging evidence suggests that FLCN may influence vesicular trafficking dynamics through interactions with cytoskeletal transport machinery. The FLCN–FNIP complex has been observed to interact with proteins regulating intracellular trafficking and membrane transport, highlighting its multifaceted role in cellular homeostasis (7, 35). Disruption of the FLCN–FNIP complex may therefore interfere with coordinated vesicular transport and lysosomal distribution. In addition to lysosomes, other endolysosomal compartments may also be affected. Endosome maturation and cargo recycling processes appear altered in several FLCN-deficient models, potentially influencing receptor turnover and the persistence of signaling cascades. For instance, altered trafficking of receptor tyrosine kinases has been proposed as a mechanism contributing to prolonged MAPK signaling in the absence of FLCN (54). Such trafficking alterations may prolong receptor signaling and thereby contribute to growth-promoting pathways activated during disease progression.

### 4.3 Implications for cellular metabolism and disease progression

The spatial reorganization of lysosomes and associated vesicular networks has broader consequences for cellular metabolism. Because lysosomes function as central hubs for nutrient recycling and metabolic signaling, their altered positioning may disturb the coordination between catabolic and anabolic pathways. In FLCN-deficient cells, these disturbances coincide with transcriptional programs driven by TFEB/TFE3 that promote lysosomal biogenesis and metabolic remodeling. The coexistence of increased lysosomal gene expression with altered spatial regulation may therefore produce an imbalanced lysosomal network characterized by expanded organelle abundance but incomplete functional coordination. Such conditions may create a permissive environment for sustained metabolic adaptation, particularly in tissues with high metabolic demands such as the kidney. Over time, these metabolic alterations may cooperate with aberrant kinase signaling and transcriptional reprogramming to facilitate cyst formation and tumorigenesis in BHD syndrome. However, the precise contribution of lysosomal spatial regulation to BHD disease progression remains incompletely defined and requires further mechanistic investigation.

## 5 Gene dosage and tissue-specific pathogenic logic

Representative genotype–phenotype correlations of recurrent and founder FLCN variants are summarized in **Table 2**, highlighting the variability in clinical penetrance and organ-specific manifestations across different mutation types (55–63).

**Table 2:** Genotype–phenotype correlations of recurrent and founder FLCN variants in Birt–Hogg–Dubé syndrome.

Variant/Class	Exon	Population (n)	Phenotype association	Evidence level	Key caveats	Key references
c.1285dupC/c.1285delC (frameshift; NMD-triggering; global hotspot)	11	Worldwide (e.g., Sweden n=352; Asia n=297; China n=100)	Pulmonary: Baseline PTX risk ~30–39% Renal: Lower RCC penetrance compared with c.779 + 1G>T CRC: c.1285dupC associated with increased CRC risk vs other variants Skin: Strong ethnicity effect (high in Caucasians; lower in Asians)	Strong (replicated in multiple large independent cohorts)	Ethnicity is major modifier of skin phenotype; ascertainment differences across cohorts	Sattler et al.2020 (55); Nordenvall et al., 2025 (56); Hu et al., 2024 (57); Namba et al., 2023 (58)
c.779 + 1G>T (splice donor; Swedish founder)	Intron 7	Sweden (n=186; n=352)	Renal: High RCC penetrance (accounts for ~61% of RCC cases in Swedish cohort) Associated with broader cancer susceptibility (RCC, CRC, melanoma)	Strong (national registry-based data)	Strong founder effect; limited generalizability outside Sweden	Lagerstedt-Robinson et al., 2022 (59); Nordenvall et al., 2025 (56)
Large intragenic deletion (exons 1–3; promoter loss; regional founder)	1–3	China (Anhui; n=76; n=100)	Pulmonary: Very high PTX risk (~91%) compared with hotspot variants (~30–39%)	Strong (replicated in regional cohorts)	Regional founder effect; lung-first referral bias limits accurate RCC risk estimation	Wang et al., 2023 (60); Hu et al., 2024 (57)
c.250-2A>G (splice acceptor; recurrent)	Intron 4	Germany (n=18 variant carriers)	Pulmonary: Increased PTX risk (~77%) vs hotspot (~37%)	Moderate (single cohort; limited variant-specific sample size)	Statistical underpowering; small variant subgroup	Sattler et al.2020 (55)
c.1300G>C (missense/splice-altering; recurrent)	11	Germany (n=22 variant carriers)	Pulmonary: Increased PTX risk (~59%) vs hotspot (~37%)	Moderate (single cohort)	Small variant-specific cohort; replication lacking	Sattler et al.2020 (55)
Exon 9 and exon 12 variant clusters (heterogeneous classes)	9, 12	Global cohorts	Pulmonary: Higher lung cyst burden (exon 9); increased PTX frequency (exons 9 & 12)	Moderate (foundational studies; widely cited)	Exon-based grouping may obscure mutation-specific effects	Toro et al., 2007 (61); Feng et al., 2025 (62)
c.769_771del (in-frame deletion; recurrent)	7	Asia (n=297)	Pulmonary: Milder PTX phenotype compared with c.1285dupC	Moderate (single large cohort)	Asian-specific dataset; requires replication	Namba et al., 2023 (58)
Putative “PTX-only” truncations (NMD-escaping; exons 13–14)	13–14	Systematic review (n=1059)	Hypothesis: renal-sparing phenotype Conclusion: Not supported; all pathogenic variants confer RCC risk	Refuted by systematic review	Age-dependent penetrance; small family size bias; statistical artifact	Matsumoto et al., 2021 (63)
Pathogenic missense variants (e.g., p.Arg362Cys; protein instability mechanism)	Various	Functional screening studies	Mechanism: Severe protein instability and proteasomal degradation; functionally equivalent to truncations	Strong (*in vitro* saturation mutagenesis and structural data)	Clinical interpretation complicated by high VUS rate	Clausen et al., 2020 (47)

Inclusion Criteria for **Table 2**: This table is not an exhaustive list of all reported FLCN variants. Variants and variant classes were selectively included based on one or more of the following criteria: (1) Epidemiological impact: High-frequency global hotspots or validated founder mutations with robust data from large cohorts or national registries; (2) Distinct phenotypic associations: Variants demonstrating unique genotype-phenotype correlations (e.g., specific pneumothorax or renal cancer risks) that inform organ-specific penetrance, regardless of cohort size; (3) Hypothesis refutation: Variant categories specifically included to address and dispel prevalent clinical misconceptions (e.g., “pneumothorax-only” variants); and (4) Mechanistic representation: Functional variant classes (e.g., missense) included to illustrate specific molecular pathogenic mechanisms rather than population frequency.

PTX, pneumothorax; RCC, renal cell carcinoma; CRC, colorectal cancer; NMD, nonsense-mediated mRNA decay; VUS, variant of uncertain significance.

Evidence Level Definition: Strong = replicated across large independent cohorts or supported by national registry-level data;

Moderate = statistically significant findings in a single cohort or limited replication.

Refuted = disproven by systematic review or meta-analysis.

Important Note: No pathogenic FLCN variant has been validated as “pneumothorax-only.” All carriers require lifelong renal surveillance.

While temporal progression explains stage-dependent signaling evolution (Section 4), gene dosage and tissue context provide an additional framework for understanding the organ-selective manifestations of BHD. Accumulating clinical and experimental evidence suggests that monoallelic and biallelic loss of FLCN generate distinct biological thresholds across kidney, lung, and skin tissues (1, 13–15). This differential sensitivity to FLCN gene dosage may help explain the marked heterogeneity observed among affected individuals and families, where patients harboring identical germline mutations may exhibit severe manifestations in one organ system while remaining relatively unaffected in others (1, 64, 65).

### 5.1 Kidney: a classical two-hit tumor suppressor model

Renal tumorigenesis in BHD generally follows a classical two-hit tumor suppressor paradigm, in which a germline mutation is followed by somatic LOH at the FLCN locus (5, 13). Biallelic inactivation has been frequently observed in microdissected renal tumors from BHD patients, supporting the notion that loss of the wild-type allele is required to initiate neoplastic transformation in the kidney (13, 66, 67).

Complete loss of FLCN function is associated with strong activation of MiT/TFE transcriptional programs, particularly within distal tubular and collecting duct epithelial compartments. Recent integrated bulk and single-cell transcriptomic analyses of BHD-associated renal tumors further support this distinct molecular signature, revealing clear differences in metabolic pathways and cellular origins compared with sporadic chromophobe renal cell carcinomas (5, 40). Kidney-specific *Flcn* knockout mouse models develop rapidly progressive cystic kidney disease, characterized by pronounced nuclear accumulation of TFEB/TFE3 and increased mTORC1 signaling in advanced lesions (45). These *in vivo* models indicate that complete genomic ablation of *Flcn* can initiate early hyperplastic changes, which may progressively evolve into solid carcinomas, partially recapitulating aspects of the human disease trajectory (68). Notably, genetic ablation of *Tfeb* markedly attenuates cyst formation, supporting a functional role for MiT/TFE activation in renal epithelial pathology (12). In addition to transcriptional activation, renal FLCN deficiency has been associated with induction of a non-canonical STAT1/2 transcriptional program, which may exert growth-modulatory effects, potentially counterbalancing proliferative signals driven by TFE3 activation (36).

Emerging evidence further refines this kidney model by revealing a nephron-segment-specific MiT/TFE driver hierarchy. Recent studies have demonstrated that in Flcn knockout mice, TFEB specifically drives cystogenesis in the loop of Henle, whereas TFE3 drives glomerular cysts (69). This tissue-specific driver hierarchy extends beyond the kidney; for instance, hepatic FLCN loss has been shown to cause cholangiocarcinoma driven specifically by TFE3 (rather than TFEB), with TFE3 depletion fully rescuing the phenotype (70). These critical observations underscore that the downstream transcriptional drivers of FLCN deficiency are not uniform but are highly dictated by tissue and cellular context.

### 5.2 Lung: haploinsufficiency and structural vulnerability

In contrast to renal tumorigenesis, pulmonary manifestations are generally thought to arise in the setting of monoallelic FLCN loss. Previous molecular analyses of lung cysts from BHD patients, relying on conventional methods, generally failed to identify somatic second-hit mutations or epigenetic silencing of the wild-type allele, supporting a haploinsufficiency-based model of pulmonary pathogenesis (42, 71). However, it is crucial to explicitly acknowledge a significant methodological constraint in the current literature: existing studies evaluating LOH have relied on conventional sequencing approaches that may underestimate low-frequency or subclonal somatic second-hit events. To our knowledge, no study has systematically assessed LOH in non-neoplastic BHD pulmonary cysts using modern sequencing approaches. Therefore, while the haploinsufficiency model represents the best current inference based on available data, it should not yet be regarded as a definitively established genetic mechanism in the lung (14). Conditional mesenchymal deletion of Flcn in murine models disrupts alveolar myofibroblast differentiation, reduces extracellular matrix components including elastin and collagen, and alters Wnt/β-catenin signaling pathways (46). Patient-derived pleural mesothelial cells carrying heterozygous FLCN mutations exhibit disruption of the E-cadherin–LKB1–AMPK axis, altered cytoskeletal organization, and increased susceptibility to apoptosis (14). These findings collectively suggest that haploinsufficiency may be sufficient to compromise mechanical integrity and tissue repair mechanisms, thereby predisposing lung tissue to cyst formation and pneumothorax without requiring full oncogenic transformation.

Recent genetic modeling has added critical nuance to this pulmonary framework. A landmark study by Cao utilizing lung mesenchyme-specific *Flcn* knockout mice demonstrated that complete biallelic loss of FLCN in mesenchymal cells causes pulmonary cyst formation via overt hyperactivation of canonical mTORC1 signaling (elevated pS6 and p4E-BP1) (72). Crucially, this cystic phenotype was substantially rescued by *Raptor* knockout or rapamycin treatment. Rather than contradicting the haploinsufficiency model, these findings complement our gene dosage framework: while human pulmonary cysts classically arise from monoallelic loss and structural disruption, the mesenchyme appears highly vulnerable to canonical mTORC1 hyperactivation upon complete biallelic FLCN loss, mirroring the two-hit pathogenic logic observed in renal tumorigenesis. Furthermore, the partial rescue observed with rapamycin in the Cao et al. study suggests that mTORC1-independent or substrate-selective mechanisms concurrently contribute to the pulmonary pathology, underscoring the complexity of tissue-specific signaling thresholds.

### 5.3 Skin: mTOR-independent haploinsufficient proliferation

Cutaneous fibrofolliculomas also appear to arise predominantly in the context of FLCN haploinsufficiency. However, the principal genetic evidence supporting this inference derives from a single study of laser-microdissected lesions from only three patients (15), in which retention of the wild-type FLCN allele was observed. These factors, including the small sample size and the limited sensitivity of the methodology employed, constrain the strength of the haploinsufficiency inference for cutaneous lesions. To our knowledge, this study did not utilize modern high-sensitivity approaches such as deep targeted sequencing or digital PCR, a limitation analogous to the evidence gap noted for pulmonary cysts in Section 5.2. Independent re-evaluation in larger patient cohorts using contemporary sequencing methods will be required to confirm that somatic second-hit events are indeed absent from fibrofolliculomas.

Despite these genetic limitations, several mechanistic observations support a model in which fibrofolliculoma formation is largely independent of canonical mTORC1 hyperactivation. First, the lack of efficacy of topical rapamycin in randomized clinical trials (16) suggests that mTORC1 inhibition alone is insufficient to drive or maintain these lesions, consistent with a FLCN-haploinsufficient state rather than an mTORC1-addicted phenotype. Second, FLCN has been implicated in the regulation of follicular stem cell differentiation, cell-cell adhesion (including E-cadherin–LKB1–AMPK axis integrity), and cAMP/PKA signaling in epithelial compartments (14, 66), suggesting that haploinsufficiency may perturb structural and differentiation programs rather than proliferative kinase cascades. Third, the strong anatomical predilection of fibrofolliculomas for sebaceous gland-rich skin indicates that local microenvironmental cues, such as sebaceous lipid signaling and androgenic hormonal input, may cooperate with FLCN haploinsufficiency to drive lesion formation (73). However, the relative contributions of MiT/TFE activation, mTORC1-independent cytoskeletal remodeling, and stromal-epithelial interactions in fibrofolliculoma pathogenesis remain to be defined.

Future studies combining conditional FLCN knockdown in follicular epithelial or sebaceous-gland cell lineages with single-cell transcriptomic profiling would be particularly informative for resolving this question and precisely mapping the mTOR-independent signaling cascades driving cutaneous BHD manifestations.

### 5.4 Integrating dosage and tissue context

**Figure 3:**
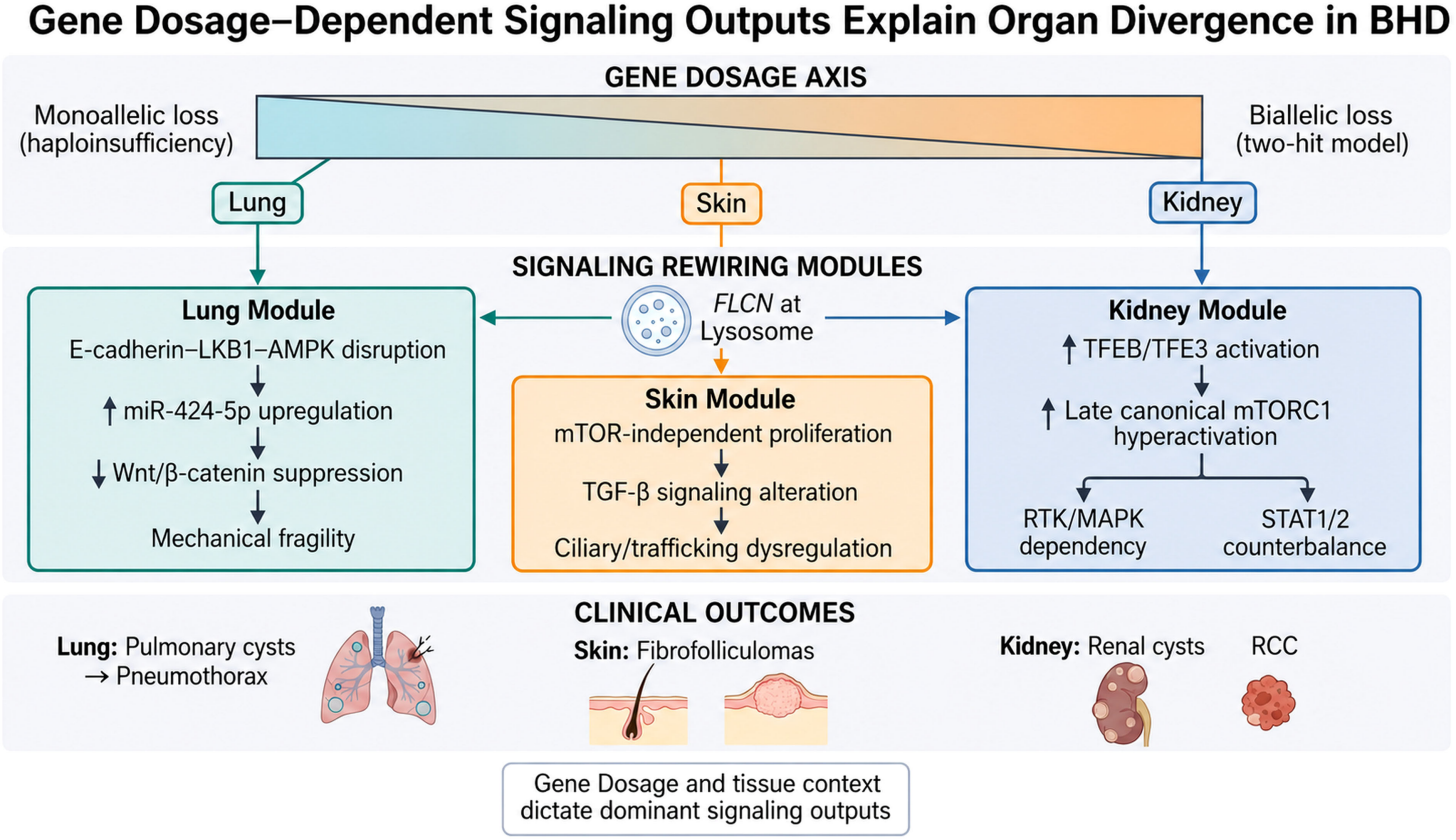
Gene dosage–dependent signaling outputs explain tissue-specific divergence in Birt–Hogg–Dubé syndrome. Integrated model linking FLCN gene dosage to tissue-specific signaling dominance and clinical outcomes. In the kidney, biallelic inactivation (two-hit model) results in complete FLCN loss, leading to immediate TFEB/TFE3 nuclear activation, subsequent transcriptional feedback amplification, late canonical mTORC1 hyperactivation, and acquisition of RTK/MAPK dependencies. A renal-specific STAT1/2 transcriptional program may partially counterbalance proliferative signaling. In contrast, pulmonary and cutaneous manifestations arise predominantly from haploinsufficiency. In the lung, monoallelic loss disrupts the E-cadherin–LKB1–AMPK axis, alters mesenchymal Wnt/β-catenin signaling, induces specific microRNAs, and compromises mechanical integrity, culminating in cyst formation and pneumothorax without requiring a somatic second hit. In the skin, haploinsufficiency promotes benign follicular proliferation through mechanisms that appear largely mTOR-independent, consistent with the limited efficacy of topical mTOR inhibition. This framework proposes that gene dosage and tissue context dictate dominant signaling outputs and divergent pathological trajectories.

In summary, these observations support a gene dosage–dependent framework in which biallelic loss in the kidney results in complete FLCN deficiency, permitting sustained MiT/TFE activation and progressive metabolic adaptation that ultimately facilitates renal tumorigenesis, whereas monoallelic loss in the lung and skin leads to a partial reduction of FLCN that disrupts adhesion, structural signaling, or differentiation pathways without uniformly triggering full oncogenic transformation. This framework accounts for why renal cancer risk generally requires a somatic second hit, whereas pulmonary cysts and fibrofolliculomas may arise in heterozygous tissues. Consequently, therapeutic strategies may need to be organ-specific, with renal disease targeting transcriptional and metabolic dependencies, while pulmonary or cutaneous manifestations may require interventions targeting structural or differentiation-related signaling pathways (**Figure 3**).

## 6 Therapeutic implications and stage-specific targeting strategies

The integrated temporal–dosage framework described above has important implications for therapeutic strategy development. Historically, therapeutic considerations in BHD have largely focused on canonical mTORC1 inhibition, largely extrapolated from early observations of mTOR pathway activation (9). However, mechanistic advances over the past decade indicate that therapeutic vulnerabilities may vary substantially depending on disease stage and tissue context (17, 18).

### 6.1 Targeting early MiT/TFE-dependent states

In early or pre-neoplastic contexts, MiT/TFE transcriptional activation appears to represent a major regulatory node following FLCN loss (10, 12). Experimental suppression of TFEB/TFE3 in FLCN-deficient renal models has been shown to reduce cystogenesis and tumor growth, supporting the functional relevance of this transcriptional axis (12). Although direct pharmacologic inhibitors targeting TFEB/TFE3 have not yet been clinically established, modulation of upstream regulators (including lysosomal signaling components and Rag GTPase interactions) has been proposed as a potential therapeutic strategy (22). These approaches remain largely investigational, and further validation in BHD-specific preclinical models will be required. In addition, emerging studies suggest potential synthetic lethality paradigms, including increased sensitivity of FLCN-deficient renal cells to PARP inhibition, which may arise from secondary impairments in homologous recombination and the BRCA1-A complex (74). It is important to emphasize, however, that this proposed synthetic lethality rests on a single preclinical study that has not yet been independently replicated. The underlying mechanistic hypothesis, which posits impaired BRCA1-A complex assembly leading to homologous recombination deficiency in FLCN-null cells, remains preliminary. Furthermore, the genomic instability profile of FLCN-deficient tumors may differ from that of classical HR-deficient cancers. Accordingly, this observation should be regarded as hypothesis-generating rather than as an established therapeutic vulnerability. Independent validation in multiple BHD-relevant model systems, including patient-derived organoids, xenografts, and isogenic FLCN-corrected controls, will be required before PARP inhibition can be considered a candidate therapeutic strategy for BHD-associated renal cell carcinoma.

### 6.2 Intermediate-stage kinase dependencies

Phosphoproteomic analyses of early-intermediate FLCN-deficient cells have identified hyperactivation of receptor tyrosine kinases, including EGFR, MET, and EPHA2, together with downstream MAPK signaling pathways (17). Functional studies further suggest that FLCN-deficient tumor cells may exhibit increased dependency on MAPK pathway signaling, raising the possibility that RTK or MEK inhibition could represent a rational therapeutic approach in selected contexts (17). Of note, such kinase dependencies are more frequently detected in transformed or chronically depleted systems but not in acute deletion models, highlighting stage-dependent therapeutic sensitivity. Furthermore, dependence on altered kinase networks may also suggest a potential role for molecular chaperone inhibitors, as FNIP co-chaperones regulate Hsp90 activity, thereby identifying Hsp90 as a possible therapeutic target for destabilizing oncogenic signaling networks in FLCN-deficient cells (37).

### 6.3 Targeting metabolic consolidation in advanced disease

Chronic FLCN deficiency manifests as upregulation of *PGC1α*, activation of HIF1α target genes, and sustained AMPK signaling (18, 33). These findings suggest that advanced tumors may rely on consolidated metabolic programs to maintain cellular growth and redox homeostasis. Potential therapeutic strategies in this context may include modulation of mitochondrial biogenesis, inhibition of glycolytic pathways, or disruption of HIF-mediated transcriptional programs. Although these approaches are conceptually supported by mechanistic data, robust preclinical validation in BHD-specific tumor models remains limited. Nevertheless, isolated clinical reports have begun to describe potential efficacy of combination therapies in advanced BHD-associated renal cell carcinomas, including cases exhibiting aggressive sarcomatoid dedifferentiation (75, 76).

### 6.4 Canonical mTORC1 inhibition: contextual role

In advanced renal cysts and tumor-derived cell lines, canonical mTORC1 activation often becomes more prominent (12, 17). Accordingly, mTOR inhibitors may retain therapeutic utility in selected late-stage contexts. However, clinical observations, particularly the lack of efficacy of topical rapamycin in cutaneous fibrofolliculomas, indicate that mTOR inhibition alone is unlikely to address all organ manifestations of BHD (16). Alternative drug-delivery approaches, such as laser-assisted topical rapamycin delivery, have also been explored to overcome skin barrier limitations in dermatological manifestations, although systemic correction of metabolic dysregulation across multiple tissues remains challenging (77, 78).

### 6.5 Organ-specific considerations

Renal disease, characterized by biallelic FLCN loss and progressive metabolic adaptation, may benefit from combination therapeutic strategies targeting MiT/TFE activity, kinase signaling dependencies, and metabolic pathways. In contrast, pulmonary and cutaneous manifestations largely arise from haploinsufficiency and structural signaling defects (14, 46). In these tissues, modulation of adhesion pathways, differentiation programs, or mechanical stress responses may represent more relevant therapeutic avenues. These observations collectively support a precision-medicine framework in which therapeutic selection may be guided by both temporal disease stage and tissue context, rather than relying on a uniform classification of BHD as an exclusively mTOR-driven disorder.

### 6.6 Conceptual outlook

As mechanistic understanding of FLCN signaling continues to improve, future research should aim to integrate longitudinal patient data with molecular staging models. Stratification of lesions according to transcriptional, kinase, and metabolic signatures may enable stage-appropriate therapeutic strategies. Contrary to the traditional view of BHD as a unidimensional disorder, emerging evidence supports a multidimensional model in which substrate-selective mTORC1 regulation, temporal adaptation, and gene dosage jointly determine the pathogenic output. Translating this conceptual framework into clinically actionable biomarkers therefore remains an important future objective.

Formal validation of this temporal model will require dedicated longitudinal experimental designs. Specifically, inducible *Flcn* deletion systems, including tamoxifen-inducible CreERT2 alleles or doxycycline-inducible shRNA/CRISPRi constructs, should be coupled with serial transcriptomic, proteomic, and phosphoproteomic profiling at defined intervals within a unified experimental system. Such longitudinal studies would clarify whether the timing and sequence of signaling transitions are conserved across tissues, and whether organ-specific kinetics contribute to the phenotypic heterogeneity of BHD. Where feasible, the complementary value of longitudinal patient-derived tissue sampling should also be explored to bridge the translational gap between experimental models and human disease progression.

## 7 Conclusions

Over the past decade, advances in structural biology, multi-omic profiling, and improved genetic models have significantly refined our understanding of BHD. It is now more accurately characterized as a lysosome-centric signaling disorder, with FLCN regulating substrate-specific mTORC1 outputs via Rag GTPase nucleotide states, rather than representing a homogenous mTORC1 hyperactivation syndrome as previously conceived (10, 22).

Within this revised framework, derepression of MiT/TFE transcription factors represents one of the earliest and most consistent molecular consequences of FLCN loss. Subsequent signaling evolution, including RTK/MAPK pathway rewiring, oxidative stress modulation, metabolic consolidation through the PGC1α/HIF axis, and delayed amplification of canonical mTORC1 signaling, unfolds in a temporally staged manner (12, 17, 18). Recognizing this temporal stratification helps reconcile previously divergent observations reported across acute deletion systems, chronic depletion models, and tumor-derived cellular contexts. This conceptual evolution aligns closely with the recently proposed “TFEopathies” framework (79), which explicitly articulates the TFEB → Rag GTPase → canonical mTORC1 feedback loop as a unifying pathogenic principle across diverse genetic contexts.

Equally important is the contribution of gene dosage to disease heterogeneity. Biallelic FLCN inactivation in the kidney supports sustained transcriptional and metabolic reprogramming compatible with tumorigenesis, whereas haploinsufficiency in lung and skin primarily perturbs structural integrity and differentiation-related pathways, without uniformly triggering full oncogenic programs (5, 14). Integrating temporal progression with gene-dosage thresholds provides a coherent explanation for the organ-selective manifestations observed in BHD.

Based on the evidence presented, a multidimensional pathogenic model emerges, in which substrate-selective signaling, temporal adaptation, and tissue-specific sensitivity jointly dictate pathogenic outcomes. This framework carries important translational implications, suggesting that therapeutic vulnerabilities may be stage-dependent and organ-specific, rather than universally centered on canonical mTOR inhibition.

Future research should prioritize longitudinal human studies integrating genomic, transcriptomic, and phosphoproteomic profiling of lesions at defined disease stages. Specifically, deep-sequencing–based reassessment of LOH in microdissected pulmonary cysts is an essential research priority; such studies would definitively clarify whether the apparent absence of somatic second-hit events reflects a genuine biological phenomenon (true haploinsufficiency) or a methodological limitation of earlier analyses. Such efforts may facilitate molecular staging of BHD-associated lesions and guide the development of precision therapeutic strategies. In parallel, continued refinement of preclinical models that recapitulate both chronic disease progression and haploinsufficient phenotypes will be essential for translating mechanistic insights into clinically meaningful interventions.

In summary, BHD provides an illustrative example of how tumor suppressor loss can generate context-dependent signaling landscapes shaped by substrate specificity, temporal dynamics, and gene dosage. Recognizing this layered signaling architecture provides a framework for reconciling mechanistic complexity with clinical heterogeneity, and may ultimately support the development of rational, stage-informed therapeutic strategies.

Furthermore, the reconciliation of acute epithelial-derived substrate-selective paradigms with chronic mesenchymal biallelic mTORC1 hyperactivation highlights an urgent need for a cell-type-resolved, dose-dependent map of FLCN signaling. Future research should prioritize spatial transcriptomics and single-cell proteomics across kidney, lung, and skin tissues at defined allelic dosages (monoallelic vs. biallelic) to precisely chart how cell lineage and gene dosage jointly dictate the transition from MiT/TFE-driven transcriptional reprogramming to canonical mTORC1 hyperactivation. Achieving this level of spatial and genetic resolution will be paramount for the development of truly personalized, organ-specific therapeutic interventions in BHD.
